# The effect of physical exercise intervention on the ability of daily living in patients with Alzheimer’s dementia: a meta-analysis

**DOI:** 10.3389/fnagi.2024.1391611

**Published:** 2024-05-31

**Authors:** Chenyu Liu, Shiying Gao, Shanshan Li

**Affiliations:** ^1^School of Sports Science, Qufu Normal University, Qufu, Shandong, China; ^2^School of Psychology, Shanghai Sport University, Shanghai, China; ^3^School of Physical Education, Sichuan University, Chengdu, Sichuan, China

**Keywords:** Alzheimer’s dementia, activities of daily living, meta-analysis, physical exercise, randomized controlled trials

## Abstract

**Objective:**

To systematically evaluate the effect of physical exercise intervention on the activities of daily living (ADL) on patients with Alzheimer’s dementia (AD) and explore the optimal exercise scheme.

**Methods:**

PubMed, EMBASE, the Cochrane Library, Web of Science, and Science Direct databases were searched from 1987 to December 2023 to collect randomized controlled trials (RCTs). Two investigators independently screened the literature and extracted data according to the inclusion and exclusion criteria. The quality of the included studies was evaluated using Cochrane Review Manager 5.3. And STATA 16.0 was used for performing the meta-analysis.

**Results:**

Fifteen randomized controlled trials were included. The results of the meta-analysis showed that physical exercise had a positive effect on the improvement of ADL in patients with AD [standardized mean difference (SMD) = 0.312, 95% confidence interval (CI 0.039–0.585), *P* = 0.02], and the difference was statistically significant. The results of subgroup analysis showed that anaerobic exercises such as strength and balance training with a medium cycle of 12–16 weeks and lasting 30–45 min each time were more ideal for the improvement of basic daily living ability of AD patients.

**Conclusion:**

Physical exercise can effectively improve activities of daily living in patients with Alzheimer’s dementia and it may be a potential non-drug treatment for AD patients.

## 1 Introduction

Alzheimer’s dementia (AD) is a degenerative disease of the central nervous system characterized by progressive cognitive, memory, behavioral, and spatial impairments (Zhao, 2020). It mainly shows a decline in cognitive ability, behavioral disorder, and a gradual decline in activities of daily living (ADL) in the clinic (Yu et al., 2006). Among them, the decline in ADL is mainly concentrated in two aspects: the decline of instrumental activities of daily living (IADL) and basic activity of daily living (BADL). A decline in instrumental ADL generally occurs in the early stages of AD. With the deterioration of the disease, patients will not be able to independently carry out basic daily activities, Liu-Seifert et al. (2016) which is the key factor leading to the loss of independence in patients with AD. Many types of dementia exist, including AD, vascular dementia, Lewy body dementia, Parkinson’s disease, mixed dementia, and frontotemporal dementia (Alzheimer’s Association, 2018). AD accounts for 60–80% of all types of dementia. The pathogenesis of AD involves many aspects, primarily genetic and environmental factors. A previous study suggests that the typical histopathology of AD includes the β-amyloid (Aβ) waterfall theory, tau protein theory, and neurovascular hypothesis (Fu and Shao, 2010). With the aging population, dementia has become one of the most serious health problems for the public and one of the main challenges for the health care system. According to a survey in 2016, there were approximately 47 million people suffering from AD worldwide, and this number is expected to double every 20 years (Barreto et al., 2015). In developed countries, the prevalence of dementia in people over 85 years of age is approximately 25–30% (Ferri et al., 2005). All the problems caused by AD force human beings to take measures to deal with this dilemma. However, the effects of medical treatment alone are not ideal. The development of new treatment methods can effectively delay disease progression.

At present, nondrug therapy has played an important supplementary role in recent years, and physical activity is an important intervention. The American College of Sports Medicine et al. (2009) stated that exercise can treat potential cognitive and motor function deterioration (Forbes et al., 2014). Some studies have also found that exercise may increase the release of protective agents such as neurotrophic factors, and affect the nervous system to improve cognitive ability (Tonoli et al., 2015). According to clinical trials, physical exercise has shown a good impact on the functional and cognitive results of patients with AD (Kwak et al., 2008; Hoffmann et al., 2016; Vidoni Perales et al., 2019), which can improve the ability of daily living in them by delaying the loss of functional independence. Physical exercise can improve functional degradation, reduce depression and anxiety, the risk of falls, and improve the patients’ independence and balance ability (Arcoverde et al., 2008).

Therefore, the conclusions of existing studies related to the effect of exercise intervention on the activities of daily living in patients with AD remain ambiguous. These results do not consistently prove that physical exercise has a clear positive effect on the ability to perform daily living tasks in patients with AD. Due to the great differences in the scope of included objectives, research areas, and measurement indicators, as well as the influence of the length of the experimental cycle, sample size, and single means of sports intervention, there is no specific and agreed upon exercise intervention implementation plan. In addition, there is no clear conclusion regarding the best protection period for exercise intervention for Alzheimer’s dementia. Due to the serious impairment of mobility in patients with severe Alzheimer’s dementia, current research is generally aimed at patients with mild to moderate Alzheimer’s dementia. The improvement in ADL outcome indicators in our study was of special significance. With the increasing number of patients with AD and limitations of existing treatment methods, it is important to determine the effectiveness of exercise interventions. This study aimed to explore whether physical exercise plays a role in the improvement of ADL of patients with AD and explore the potential biological mechanisms of physical activity on ADL of patients with AD to determine the circumstances under which physical exercise should be added to the treatment plan, and formulate a more reasonable exercise plan to provide safer and more effective guidance for improving the daily living ability of patients with AD.

## 2 Methods

### 2.1 Eligibility criteria

The methods and results of this study are reported in accordance with Preferred Reporting Items for Systematic Evaluation and Meta-analysis guidelines. The inclusion and exclusion criteria are listed in Table 1.

**TABLE 1:** Inclusion and exclusion criteria.

	Inclusion criteria	Exclusion criteria
Study type	RCT published in English in journals or conference proceedings, and the full text can be obtained.	Non-RCT experiment, review literature, non-English literature, study with lack of data or unable to obtain the full text, repeated publication and literature of poor evaluation quality.
Population	Patients with Alzheimer’s dementia include mild, moderate and severe patients.	The subjects were not patients with Alzheimer’s disease, such as Parkinson’s disease and Animal experiments.
Intervention	Aerobic exercise, anaerobic exercise and physical exercise combined with a variety of exercise contents	Non exercise intervention or mixed intervention
Outcome	Indicators used in the evaluation of activities of daily living: ① Alzheimer’s disease collaborative scale of activities daily living (ADCS-ADL) scale; ② Activities of daily living (ADL) scale; ③ Basic activities of daily living (BADL) scale; ④ Instrumental activities of daily living (IADL) scale.	The indicators not accessing activities of daily living scale, such as Alzheimer’s disease assessment scale or Mini-Mental State Examination (Zhu et al., 2015).
Control	The control group was treated with non-exercise intervention or no intervention at all	The control group received exercise intervention.

### 2.2 Retrieval strategy

PubMed, EMBASE, Cochrane Library, Web of Science, and Science Direct databases were searched between 1987 and December 2023 to collect randomized controlled trials (RCTs) on the effect of physical exercise on the ability of daily living of patients with AD. The strategy was formulated based on the medical subject title and the Boolean operator, and the retrieval adopted a combination of subject words and free words. The search terms included “physical exercise,” “Alzheimer’s dementia,” “activities of daily living,” and “randomized controlled trial.” Taking PubMed as an example, Box 1 shows the specific retrieval strategy.

BOX 1 **PubMed retrieval strategy.**

#1 Physical exercise [MeSH Terms]

#2 Exercises OR Physical Activity OR Exercise, Physical OR Acute Exercise OR Exercise, Isometric OR Exercise, Aerobic OR Exercise Training

#3 #1 OR #2

#4 Alzheimer Disease [MeSH Terms]

#5 Alzheimer Dementia OR Dementia, Senile OR Alzheimer-Type Dementia (ATD) OR Alzheimer Type Senile Dementia OR Primary Senile Degenerative Dementia OR Alzheimer Sclerosis OR Alzheimer Syndrome OR Senile Dementia, Alzheimer Type OR Acute Confusional Senile Dementia OR Dementia, Presenile OR Alzheimer Disease, Late Onset OR Alzheimer’s Disease, Focal Onset OR Familial Alzheimer Disease (FAD) OR Alzheimer Disease, Early Onset

#6 #4 OR #5

#7 Activities of daily living [MeSH Terms]

#8 ADL OR Activity, Daily Living OR Chronic Limitation of Activity

#9 #7 OR #8

# 10 “randomized controlled trial”[pt] OR “controlled clinical trial”[pt] OR randomized[tiab] OR placebo[tiab] OR “drugtherapy”[sh] OR randomly[tiab] OR trial[tiab] OR groups[tiab]

#11 #3 AND #6 AND #9 AND #10

### 2.3 Literature screening and data extraction

Two researchers independently screened the literature according to the inclusion and exclusion criteria and extracted the data. Differences were resolved through discussion or consultation with senior researchers. When the study met the inclusion criteria, following data were collected: ① basic information of the included study; ② the mean number of samples in each group and the characteristics of the study area, including the baseline sample number, sex, etc.; ③ details of intervention content; and ④ the mean and standard deviation of outcome indicators after intervention.

### 2.4 Risk assessment of bias

Two researchers evaluated the methodological quality of 15 included studies from seven aspects (random sequence generation, allocation concealment, blinding, detection bias, incomplete outcome data, selective reporting, and other bias) according to the bias risk evaluation criteria for RCTs in the Cochrane manual. Each aspect was evaluated for “low risk,” “unclear,” and “high risk;” studies with 6 to 7 frequencies of “low risk” were classified as low bias risk, studies with 4 to 5 frequencies of “low risk” were classified as moderate bias risk, and studies with less than 4 frequencies of “low risk” were classified as high bias risk. Two researchers independently reviewed each other. When there were differences, a third researcher jointly discussed and decided whether to include them.

### 2.5 Statistical analysis

Stata16.0 (Stata- Corp), was used for the meta-analysis of the data of outcome indicators with a random effect model of D-L method. Since the outcome indicator is a continuous variable, and there are differences in the scale of “ability of daily living” included in the included studies, the standardized difference standardized mean difference (SMD) and its 95% confidence interval (CI) were used as the indicators of the effect size. The I^2^ statistic was used to determine the size of heterogeneity. Heterogeneity was considered small with I^2^ of 0–40%, moderate with I^2^ of 30–60%, great with I^2^ of 50–90%, and significant with I^2^ of 75–100%. The means and standard deviations after intervention were extracted from the study. If the mean and standard deviation are not provided in the study, we will check whether they can be calculated with appropriate data. The effect size was divided into small (less than 0.2), medium (0.2–0.8) and large (more than 0.8). If there was great heterogeneity between research results, the source of heterogeneity should be further explored through subgroup and sensitivity analyses. The Egger test was performed using Stata16.0, to evaluate small study effects and publication bias. Patients or the public were not involved in the design, or conduct, or reporting, or dissemination plans of our research.

## 3 Results

### 3.1 Study retrieval

**FIGURE 1:**
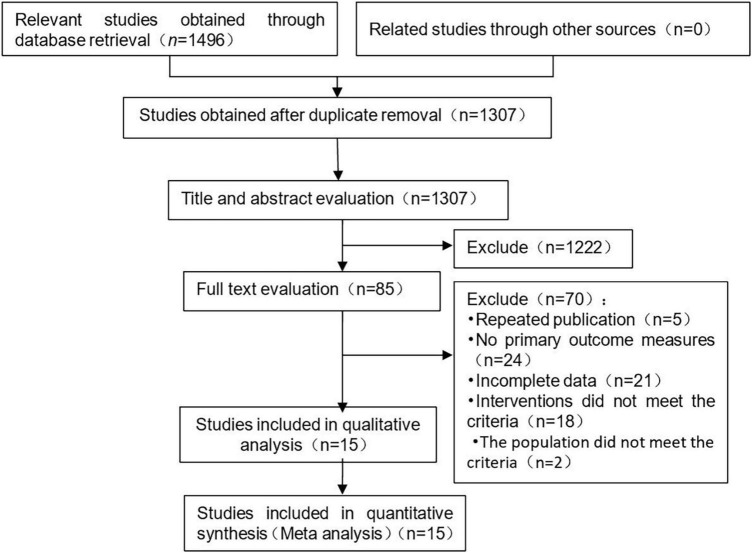
Description of the selection process for included studies.

A total of 1,496 literatures were preliminarily retrieved, and 189 published articles were eliminated. 1,222 literatures were preliminarily excluded after reading the title and abstract, and 70 articles were eliminated after full-text reading. Fifteen studies were included in this meta-analysis. The process and results of literature screening are shown in Figure 1.

### 3.2 Study characteristics and risk of bias within studies

Fifteen studies with 20 trials were included in this study. The characteristics of the included studies are presented in Table 2. A total of 1,420 patients with AD participated in the study, including 768 patients from the experimental group and 652 patients from the control group. The average age of the included patients was 65–85 years. The sample sizes ranged from 14 to 375. Patients participated in resistance training, strength training, flexibility and balance training and some moderate-intensity aerobic exercise. These intervention programs vary widely, ranging in frequency from 2 to 6 sessions per week and varying from 30 to 60 min per exercise, the intervention cycle ranged from 4 to 48 weeks. The outcome indicators included the ADL, IADL, and BADL scales.

**TABLE 2:** Study characteristics.

References	Sample size (T/C)	Age	Intervention content	Cycle (week)	Duration (min)	Frequency (times/week)	Outcome
Maltais et al., 2019	40/45	≥65	Combination training	24	60	2	ADCS-ADLIADLBADL
Hoffmann et al., 2016	102/88	50–90	Aerobic training	16	60	3	ADCS-ADL
Maci et al., 2012	7/7	72.6 ± 9.5	Combination training	12	60	5	IADLADL
Vreugdenhil et al., 2012	20/20	51–89	Combination training	16	30	>1	IADLADL
Lamb et al., 2018a	251/124	77 ± 7.9	Aerobic training	16	60	2	BADL
Littbrand et al., 2009	43/48	≥65	Anaerobic training	13	45	5	ADL
Oliveira et al., 2021	16/19	≥65	Anaerobic training	16	60	3	ADL
Rolland et al., 2007	56/54	82.8 ± 7.8	Combination training	48	60	2	ADL
Kwak et al., 2008	15/15	≥60	Aerobic training	48	30–60	2–3	ADL
Kampragkou et al., 2017	15/15	≥65	Combination training	12	30	3	ADL
Venturelli et al., 2011	11/10	84+5	Aerobic training	24	30	4	ADL
Shaw, 2021	14/20	82.21 ± 6.62	Combination training	8	45	3	ADCS-ADL
Nascimento et al., 2014	14/16	76.8 ± 6.8	Aerobic training	24	60	3	IADL
Elisabeth et al., 2017	78/82	81.1 ± 7.7	Combination training	4	30	5	ADL
Pitkänen et al., 2019	86/89	77.7 ± 8.2	Anaerobic training	Unclear	45	6	ADCS-ADL; BADL

T, experimental group; C, control group. ADL, activities of daily living scale; ADCS-ADL, Alzheimer’s disease collaborative daily ability scale; BADL, basic activities of daily living scale; IADL, instrumental activity of daily living scale.

**FIGURE 2:**
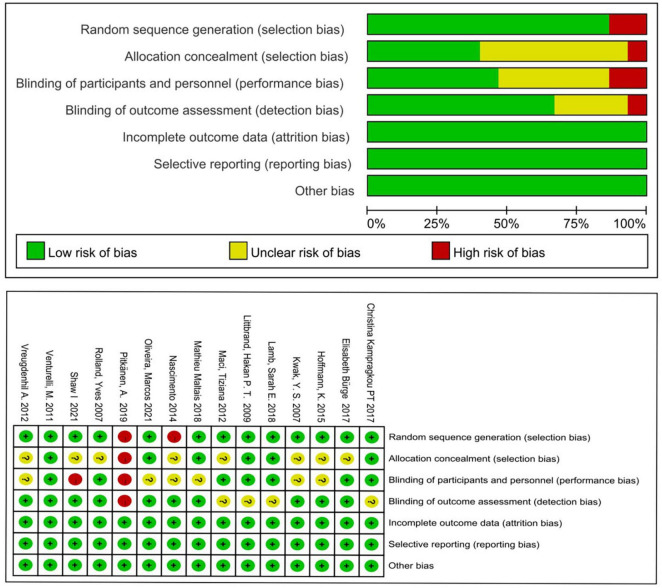
Assessment of the risk of bias for included studies.

The results of the risk of bias assessment are shown in Figure 2. Five of the 15 studies reported outcome indicators after follow-up. As shown in Figure 2, among the 15 included studies, 13 studies described the generation of random sequences, 6 studies described the method of allocation and concealment, 7 were double-blinded, 10 used the evaluation blind method, and all the studies reported complete data and reasons for loss of follow-up. Overall, the included studies were of high quality. One study (Pitkänen et al., 2019) was assessed as high-risk, so the data of this study were eliminated from further meta-analysis.

### 3.3 Meta-analysis

#### 3.3.1 Heterogeneity test

**FIGURE 3:**
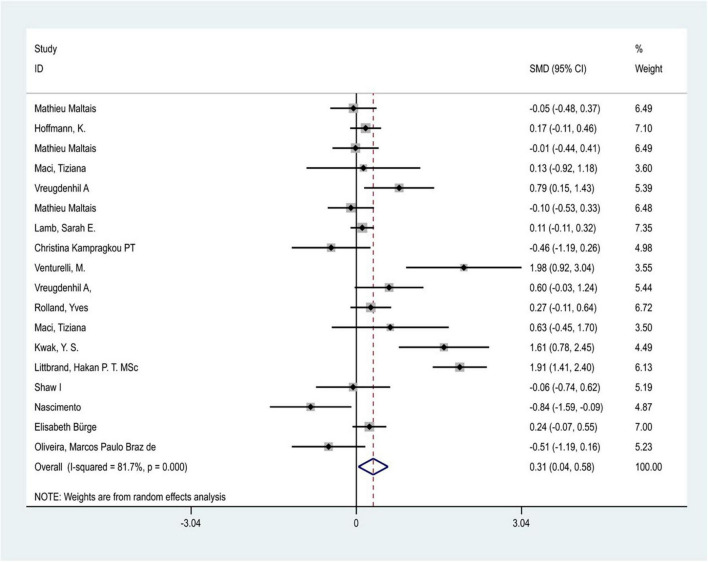
Heterogeneity of the included studies.

Considering the great heterogeneity among the included studies (*P* < 0.05, I^2^ > 50%), the meta-analysis was performed using a random-effects model. A total of 18 trials in 15 included studies (one study was removed because of a high risk of bias) evaluated the effect of physical exercise intervention on the ability of daily living in patients with Alzheimer’s dementia. The results of the meta-analysis showed that there was significant heterogeneity between studies (I^2^ = 81.7%, *P* < 0.05), and physical exercise had a positive effect on the improvement of ADL in patients with AD [SMD = 0.312,95% CI (0.039–0.585) *P* = 0.02] (Figure 3). In addition, five included studies reported follow-up outcomes, and the follow-up time ranged from 6 to 12 months. The results showed that physical exercise had no significant effect on the improvement of ADL in patients with AD during the follow-up period [SMD = 0.064, 95% CI (−0.113–0.241), *P* = 0.47].

#### 3.3.2 Sensitivity analysis and publication bias test

**FIGURE 4:**
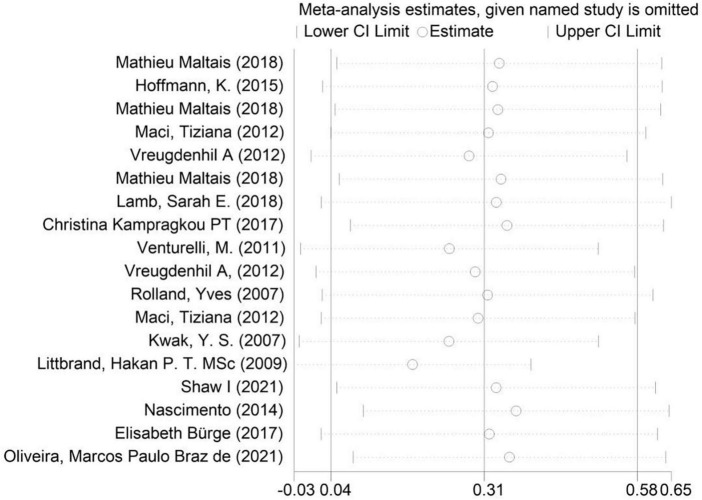
Sensitivity analysis of the included studies.

**FIGURE 5:**
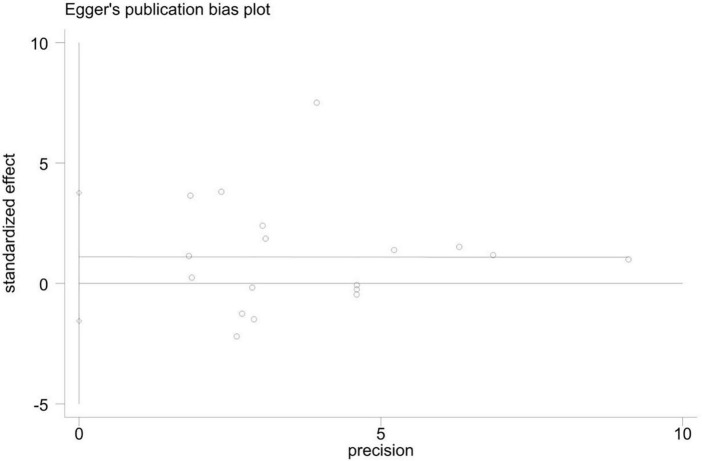
Publication bias of the included studies. Graphics created using Adobe Illustrator 2021.

As shown in the sensitivity analysis (Figure 4), the 95% confidence interval is 0.04–0.58 and the estimated value of the middle point is 0.31; the value of each article fluctuates up and down within the estimated value of the point and all of them are within the confidence interval. The Egger test was used to examine publication bias (Figure 5). The results showed no obvious publication bias, indicating that our study had high reliability and reference values (*P* = 0.392 > 0.05, 95% CI included 0).

#### 3.3.3 Subgroup analysis

Through subgroup analysis of the region (Table 3), it was found that studies in Europe have significant heterogeneity in the effect of improving ADL in patients with AD (I^2^ = 89.7%), indicating that it has an impact on the relationship between physical exercise and the improvement of ADL in patients with AD. Studies in Oceania (*d* = 0.695, *P* = 0.003) found that exercise intervention had a better effect on improving ADL in patients with AD than in Europe (*d* = 0.517, *P* = 0.025) and North America (*d* = −0.417, *P* = 0.000). However, there were only two included studies from Oceania that were not representative.

**TABLE 3:** Subgroup meta-analysis of the effect of physical exercise intervention on the ability of daily living of patients with Alzheimer’s disease.

Subgroup	Numbers of included studies	Heterogeneity		The result of meta-analysis		
		*I* ^2^	*P*-value	SMD (95%CI)	*Z*-value	*P*-value
**Study location**						
Oceania	2	0.0%	0.686	0.695 (0.243, 1.147)	3.01	0.003
Europe	7	89.7%	0.000	0.517 (0.065, 0.968)	2.24	0.025
North America	7	1.1%	0.416	−0.417 (−0.633, −0.201)	3.79	0.000
**Age (years)**						
60–75	8	92.0%	0.000	0.156 (−0.446, 0.758)	0.51	0.612
≥75	10	63.9%	0.003	0.295 (0.010, 0.580)	2.03	0.043
**Intervention**						
Aerobic	8	81.6%	0.000	0.407 (−0.017, 0.830)	1.88	0.060
Anaerobic	2	96.9%	0.000	0.709 (−1.661, 3.079)	0.59	0.558
Combination	8	58.4%	0.019	−0.084 (−0.360, 0.191)	0.60	0.550
**Frequency (day)**						
≥3	10	76.2%	0.000	0.212 (−0.191, 0.614)	1.03	0.302
<3	8	90.8%	0.000	0.273 (−0.210, 0.755)	1.11	0.268
**Cycle (week)**						
4–8	2	0.0%	0.475	0.167 (−0.115, 0.450)	1.16	0.246
12–16	9	86.0%	0.000	0.386 (−0.065, 0.836)	1.68	0.093
24–48	7	86.9%	0.000	0.136 (−0.425, 0.697)	0.48	0.634
**Duration (min)**						
30–45	8	87.6%	0.000	0.792 (0.184, 1.400)	2.55	0.011
60	10	63.4%	0.003	−0.141 (−0.380, 0.098)	1.16	0.247
**Outcome**						
ADCS-ADL	3	64.4%	0.060	−0.096 (−0.524, 0.332)	0.44	0.662
IADL	4	77.0%	0.005	−0.093 (−0.799,0.613)	0.26	0.796
BADL	6	92.9%	0.000	0.564 (−0.186, 1.314)	1.47	0.140
ADL	5	74.6%	0.003	0.366 (−0.127, 0.860)	1.45	0.146
**Follow up duration (months)**						
6	6	37.0%	0.160	0.051 (−0.148, 0.251)	0.51	0.613
12	6	83.2%	0.000	0.021 (−0.378, 0.419)	0.10	0.919

ADL, activities of daily living scale; ADCS-ADL, Alzheimer’s disease collaborative daily ability scale; BADL, basic activities of daily living scale; IADL, instrumental activity of daily living scale; SMD, standardized mean difference.

The subgroup analysis of age showed that the subgroup of patients aged 60–75 years had significant heterogeneity (I^2^ = 92%). Physical exercise was found to have a better effect on the ADL of patients with AD over 75 years of age (*d* = 0.295, *P* = 0.043).

Through the subgroup analysis of intervention content, the subgroups of anaerobic exercise intervention (I^2^ = 96.9%) and aerobic exercise intervention (I^2^ = 81.6%) showed significant heterogeneity. Among them, anaerobic training (*d* = 0.709, *P* = 0.558) showed a better effect on improving the ADL of patients with AD than aerobic training (*d* = 0.407, *P* = 0.060) and combination training (*d* = −0.084, *P* = 0.550).

In subgroup analysis of intervention frequency, the subgroup with an intervention frequency of more than 3 days a week showed great heterogeneity (I^2^ = 76.2%), and the subgroup with an intervention frequency of less than 3 days a week (I^2^ = 90.8%) showed significant heterogeneity. Among them, an intervention frequency of less than 3 days a week has a better effect on the improvement of ADL in patients with AD.

Through subgroup analysis of the intervention cycle, subgroups at 12–16 weeks (I^2^ = 86.0%) and 24–48 weeks (I^2^ = 86.9%) showed significant heterogeneity. Exercise with an intervention cycle of 12–16 weeks had a greater effect on the improvement of ADL in patients with AD (*d* = 0.386).

Through subgroup analysis of intervention duration, the subgroup of 30–45 min showed significant heterogeneity (I^2^ = 87.6%), and the intervention duration of 30–45 min had a better effect on the improvement of ADL in patients with AD (*d* = 0.792, *P* = 0.011).

In subgroup analysis of the scale used to evaluate the ability of daily living, the BADL subgroup showed significant heterogeneity (I^2^ = 92.9%) and had a better improvement in ADL.

By grouping the follow-up data reported in the 4 included studies, it was found that the subgroup of 12-month follow-up results showed great heterogeneity (I^2^ = 83.2%), and there was no significant difference in the intervention effect before and after follow-up.

Based on the above analyses, it was concluded that 12–16 weeks of moderate cycle and 30–45 min of anaerobic exercise, such as strength and balance training, are more ideal for the improvement of ADL of AD patients.

## 4 Discussion

The purpose of this study was to systematically evaluate the effect of physical exercise intervention on ADLon patients with AD and explore the optimal exercise scheme. According to the established inclusion and exclusion criteria, 15 RCT studies were included in the meta-analysis. The results showed that physical exercise had a positive effect on the improvement of ADL in patients with AD [SMD = 0.312, 95% CI (0.039–0.585), *P* = 0.02] and had a better effect on the improvement of BADL [SMD = 0.564,95% CI (−0.186 to 1.134]. The degree of benefit depends on the type of physical exercise, exercise cycle, and exercise duration. The results showed that physical exercise had no significant effect on the improvement of ADL in AD patients before and after follow-up (SMD = 0.064, 95% CI (−0.113 to 0.241) *P* = 0.47) The subgroup analysis showed that 12–16 weeks of moderate cycle and 30–45 min of anaerobic exercise, such as strength and balance training, were more ideal for the improvement of the basic daily living ability of AD patients. However, the follow-up data showed that there had no significant effect in AD patients after stop exercising, which indicates that this intervention may require long-term persistence.

Brain derived neurotrophic factor (BDNF) is a neurotrophin that promotes the survival of neurons and is essential for regulating memory function (Loprinzi and Frith, 2019). It has been proved that the impact of exercise on brain plasticity is associated with the action of brain-derived neurotrophic factor (de Melo Coelho et al., 2014). Exercise can increase the concentration of brain-derived neurotrophic factor, promote nerve regeneration, improve nervous system, so as to improve the brain structure involved in cognition (Pereira et al., 2007; Gomez-Pinilla et al., 2011; Liu et al., 2011), reduce the degree of dementia in patients with AD and further improve their ability of daily living. The internal mechanism of its influence is mainly the cross dialogue between muscle tissue and other organs. Cytokines released into the blood by muscle contraction reach the brain, which can promote the production of brain-derived neurotrophic factors (Delezie and Handschin, 2018). Studies have shown that lactic acid can activate nuclear factors- kappaB (NF- kB) signaling pathways promote neuronal survival (Zhou et al., 2018). In addition, hippocampus plays an important role in neural plasticity. Atrophy of the hippocampal and entorhinal cortex has been shown to predict conversion to AD. It is important to assess atrophy of the hippocampus and entorhinal cortex to understand the AD pathology (Yoshida et al., 2012). Exercise can increase the volume of gray matter and white matter in the prefrontal and temporal cortex of the hippocampus, enhance hippocampal neurogenesis, reduce the aggregation of tau protein, and improve the ability of daily living in patients with AD (Ahlskog et al., 2011). Some studies showed that long-term exercise can increase the volume of the hippocampus by 2% and delay the development of dementia (Colcombe et al., 2006), indicating that the beneficial degree of intervention also depends on the length of exercise cycle. This is consistent with the results of this study, which found that the 12–16 weeks exercise cycle is ideal. The biological mechanism of physical exercise in Alzheimer’s dementia is also reflected in the improvement of related immune system function at the cell and molecular levels. Previous research has found that physical activity inhibits the decline of mitochondrial and immune system functions associated with Alzheimer’s dementia (McDermott et al., 2013). In addition, studies have shown that exercise can improve cerebral blood flow in the frontal and temporal lobes, increase the oxygen supply, and improve cerebral circulation (Lista and Sorrentino, 2010). In addition to these benefits, exercise can also play an anti-inflammatory role and improve oxidative damage to the brain. Chronic inflammation and oxidative damage are important causes of AD. Studies have found that moderate intensity exercise can promote the reduction of NLRP3 inflammation (Khakroo Abkenar et al., 2019), improve mitochondrial function, promote redox balance, and slow down the development of AD (Bernardo et al., 2016). From the perspective of skeletal muscle, physical exercise can improve the quality of skeletal muscle, improve the endurance and flexibility of skeletal muscle, reduce cardiovascular stress response, and provide strength to patients for lower body activities (Jin et al., 2020). The biological mechanism of the intervention effect of physical exercise on the ability of daily living in patients with AD has been confirmed by a large number of experimental studies; especially the effect of long-term physical exercise on AD, which is more obvious, as well as sufficient evidence of changes to the pathological mechanism. Compared with previous relevant studies, the subjects included in this study were patients with mild-to-moderate AD. The scope of exercise intervention measures was wider for later comparison, and the outcome indicators were more comprehensive to ensure the reliability of the final results.

An increasing number of scholars have begun to pay attention to the effect of physical exercise intervention on the treatment of AD and to further analyze the improvement of function of patients with AD. The results of this study showed that exercise therapy can significantly improve the scores of various ADL scales, reduce the risk of hospitalization, slow down the progress of AD, and improve the quality of life of patients to a certain extent. Previous studies have also shown that regular exercise can make AD patients more independent and improve their cognitive and daily living ability (Garuffi et al., 2013; Holthoff et al., 2015; Toots et al., 2016). However, due to differences in intervention contents, intervention cycles, scale, inclusion, and exclusion criteria, studies have produced different results and benefits. According to Lamb et al. (2018b), there is insufficient evidence that moderate-to-high-intensity structured exercise can improve patients’ overall activities of daily living. Similar results can be found in another study (Hoffmann et al., 2016; Kampragkou et al., 2017) as there was no change in ADL score after the intervention of aerobic exercise among AD patients. The results from a group of randomized controlled experiments showed that the ADL of Alzheimer’s dementia patients in the exercise group was improved through exercise, which significantly reduced the burden on caregivers, while the burden of caregivers in the control group doubled (Lowery et al., 2014). Vreugdenhil et al. (2012) designed a 4-month randomized controlled trial in which 40 community patients with AD and their informal caregivers were randomly assigned to the exercise group or the control group (routine treatment) to implement a family exercise program. The results showed that the instrumental activities of daily living (IADL) scale increased by 1.6 points and the life independence of patients with AD was significantly enhanced. Rolland et al. (2007) implemented a 1-year group exercise program for 134 patients with mild-to-severe AD. The results showed that, compared with patients receiving routine medical care, the rate of decrease in the ADL score in the exercise group was lower than that in the control group. However, other studies have shown that physical exercise has no evident positive effect on ADLs in patients with AD. Maltais et al. (2019) conducted a structured exercise intervention on 91 patients with AD for 6 months. After the exercise intervention, the ADL score of AD patients in the exercise intervention group decreased significantly, and the IADL score decreased the most, whereas the participants in the social intervention group maintained their original level. Burge et al. (2017) conducted a randomized controlled trial on patients with moderate to severe dementia to measure the effect of physical exercise program (strengthening, balance and walking) on ADL score. The results showed that sports could delay the loss of mobility but had no significant effect on the total ADL score.

A recent umbrella review by Lopez-Ortiz et al. (2023) synthesizes existing meta-analyses on the effects of physical activity and exercise interventions on AD. While this review provided a broad comparison of related studies, it also highlighted a gap in the literature regarding the impact of physical exercise specifically on ADL in Alzheimer’s patients. Notably, only two prior meta-analyses (Rao et al., 2014; Zhou et al., 2022) have focused on this aspect, neither of which included a sensitivity analysis to assess the robustness of their findings. Our meta-analysis addresses this gap by incorporating a larger dataset, including studies from both earlier and recent publications, thereby offering a more robust and comprehensive analysis. In this study, through a meta-analysis of 15 high-quality studies, it was concluded that physical exercise had a positive impact on ADL on patients with Alzheimer’s dementia. Our inclusion of sensitivity analyses further strengthens our findings, enhancing their reliability and applicability in clinical settings. In contrast to other studies, the outcome included in this study had the longest follow-up period. Through analysis and comparison of the data before and after follow-up, it was found that the protective effect of exercise on the ability of daily living in patients with AD may be short, and a period of lack of exercise may lead to a decline in ADL.

Furthermore, as Lopez-Ortiz et al. (2023) suggest, there remains a need to define the exercise parameters (type, volume, intensity) that yield the most significant benefits for ADL in Alzheimer’s patients. Through subgroup analysis, a more specific exercise intervention scheme was formulated according to the combined effect value. Our study contributes directly to this area by evaluating these variables and providing actionable insights that can guide future exercise recommendations for this population.

This study had some limitations. The research results may be affected by the comprehensiveness of the retrieved literature, including the means of intervention, intervention cycle, intervention frequency, sample size, severity of AD, measurement scale, and other social factors, which may have resulted in great heterogeneity among the included studies. In addition, AD is a multidimensional disease, which requires a large number of experiments to evaluate the results. The number of studies included in this review was limited. Only five included studies reported follow-up results; therefore, it is difficult to evaluate the long-term efficacy of exercise intervention. In addition, since the population included in this study was of patients with mild to moderate AD, whether physical exercise is suitable for all patients with AD and whether the exercise ability of the subjects will affect the results needs to be considered. After the sensitivity analysis and exclusion analysis of the literature, it was found that Kwak et al. (2008), Littbrand et al. (2009), Venturelli et al. (2011), and Nascimento et al. (2014) have a large bias. Therefore, a meta-analysis of the remaining RCTs after exclusion showed that heterogeneity was significantly reduced (I^2^ = 22.0%, *P* = 0.215). The above four articles were likely to be the source of heterogeneity. In these studies, the subjects were patients with mild to moderate dementia of AD, which was different from other studies, or all the subjects were women, and the Preffer tool activity questionnaire used in the measurement index may be the reason for the heterogeneity (Nascimento et al., 2014). This study was not registered, and a protocol was not prepared.

According to this meta-analysis, relevant research on physical exercise improving AD shows that the experimental studies of single exercise content were in the majority, and there were few studies of comprehensive exercise intervention. More high-quality research needs to be collected in future, and the results of the meta-analysis needs to be updated and the exercise program for patients with different degrees of AD need to be constantly optimized.

To summarize, physical exercise has a positive effect on the ability to perform daily living in patients with AD. For patients with mild to moderate AD, 12–16 weeks of a moderate cycle and 30–45 min of anaerobic exercise, such as strength and balance training, are ideal for the improvement of basic daily living ability. Further studies are needed to investigate the specific details of exercise interventions and formulate a more accurate and effective exercise plan for patients with AD.

## 5 Conclusion

The results showed that physical exercise had a positive effect on the improvement of ADL in patients with AD [SMD = 0.312,95% CI (0.039–0.585), *P* = 0.02] and had a better effect on the improvement of BADL [SMD = 0.564,95% CI (−0.186 to 1.134]. The degree of benefit depends on the type of physical exercise, exercise cycle, and exercise duration. The results showed that physical exercise had no significant effect on the improvement of ADL in AD patients before and after follow-up (SMD = 0.064,95% CI (−0.113 to 0.241) *P* = 0.47). The subgroup analysis showed that 12–16 weeks of moderate cycle and 30–45 min of anaerobic exercise, such as strength and balance training, were more ideal for the improvement of the basic daily living ability of AD patients. However, the follow-up data showed that there had no significant effect in AD patients after stop exercising, which indicates that this intervention may require long-term persistence.

## Author contributions

CL: Writing – review and editing, Writing – original draft, Methodology, Formal analysis. SG: Writing – review and editing, Writing – original draft, Methodology, Data curation. SL: Writing – review and editing, Writing – original draft, Supervision, Methodology, Conceptualization.
